# How ethics committees and requirements are structuring health research in the Philippines: a qualitative study

**DOI:** 10.1186/s12910-021-00653-z

**Published:** 2021-07-01

**Authors:** Gideon Lasco, Vincen Gregory Yu, Lia Palileo-Villanueva

**Affiliations:** 1 Development Studies Program, School of Social Sciences, Ateneo de Manila University, 4th Floor, Ricardo & Dr. Rosita Leong Hall, University Road, Katipunan Ave., Loyola Heights, Diliman, 1108 Quezon City, Philippines; 2 Department of Anthropology, University of the Philippines Diliman, Quezon City, Philippines; 3 College of Medicine, University of the Philippines Manila, Manila, Philippines

**Keywords:** Research ethics committees, Institutional review boards, Research ethics, Health research, Research inequity, Philippines

## Abstract

**Background:**

The last few decades have seen the rising global acknowledgment of the importance of ethics in the conduct of health research. But research ethics committees or institutional review boards (IRBs) have also been criticized for being barriers to research. This article examines the case of the Philippines, where little has been done to interrogate the health research and IRB culture, and whose circumstances can serve as reflection points for other low- and middle-income countries.

**Methods:**

Semi-structured interviews were conducted from July to October 2020 to elicit health researchers’ perspectives and experiences regarding IRBs and the ethics approval process in the country, as well as counterpoint narratives from researchers who have also worked for IRBs.

**Results:**

Across the fields of clinical, public health, and social science research, the issue of ethics review revealed itself to be foremost an issue of inequity. IRB processes serve as a barrier for those outside the academe; those belonging to institutions, cities, or entire regions without their own accredited IRBs; and researchers working independently, without ample budget, or on highly specialized topics—more so for non-clinical researchers who must grapple with the primarily biomedical framework of most IRBs. Consequently, the research landscape invariably favors those with the resources to do research, and researches that tend to attract funding.

**Conclusion:**

The broader challenge of equity in health research will entail more fundamental reforms, but proximal interventions can be done to make the ethics approval process more equitable, such as enhancing institutional oversight, regulating IRB fees, and enabling a more supportive and welcoming environment for early-career, student, independent, and non-clinical health researchers. This article ends by reflecting on the implications of our findings toward the larger research culture.

**Supplementary Information:**

The online version contains supplementary material available at 10.1186/s12910-021-00653-z.

## Background

The past few decades have witnessed an increasing acknowledgment of the importance of ethics in the conduct of human research. Consequently, professional organizations and academic institutions around the world have organized ethical guidelines and review committees (here on alternately referred to as ethics boards or institutional review boards [IRBs]) to ensure researchers’ adherence to ethical principles. As such, securing ethics approval has become essential to the initiation, funding, and publication of health research.

But IRB processes have also been criticized for being a barrier to research. Examining two multi-center Australian researches, Barnett et al. found high costs—both in terms of time and money—in obtaining ethics approval [1]. Kendall and Halliday highlighted particular issues faced by qualitative researchers, including the challenges posed by a “biomedical research ethics paradigm” to researchers with “a social justice agenda.” [2] (p. 308) Summarizing concerns raised by existing scholarship, Nicholls et al. wrote:

While few would disagree with the general need for ethics review, existing review processes are often criticized; common complaints include the amount of paperwork required, inconsistency of decisions between review boards, and suggestions that ethics review systems may not be equipped to properly review specific types of research [3].

Nicholls et al. further underscored the absence of “gold standards against which to evaluate research ethics review processes”; and, crucially for the Philippine context, how “there has been little in the way of published research on the subject of assessment of research ethics review.” [3] In fact, a quick PubMed search, coupled with our preliminary library research, revealed no studies dealing with this topic in the Philippines.

Following the above and other researches, we set out to examine the ethics approval process in the Philippines according to these three questions:

1. How are demands for ethical research structuring the health research landscape?;
2. What are the experiences and particular challenges of Filipino health researchers with regards to IRBs?; and
3. How exactly do researchers from specific disciplines (e.g. clinical researchers versus social scientists) feel about ethics and IRBs?

Our study builds a preliminary knowledge base—and not necessarily an exhaustive picture—for this particular topic in the Philippines. Through qualitative interviews that elicited the perspectives and experiences of Filipino researchers themselves, as well as people involved in Philippine IRBs, this study charts the ethics approval process in the country and identifies contemporary barriers and facilitators along the way. Building on this data, we offer policy recommendations for academic institutions, IRBs, and government bodies that not only speak to the situation of health research in the Philippines, but are also relevant to other contexts, especially in low- and middle-income countries where researchers and institutions alike have likewise adapted to the demands of ethics in research.

### Ethics review in the Philippines

Relative to the Global North, the institutionalization of research ethics processes in the Philippines is a fairly late trend. In the late 1980s, for example, IRBs were already present in over 60 percent of hospitals in the United States [4]; while by 2000, “more than 95% of Portuguese hospitals had established” an IRB [5] (p. 485).

In contrast, in the 1980s the Philippines was only seeing the formation of a National Ethics Committee, tasked to “promote ethics review in health research.” [6] Within that decade, the only major academic or research institution in the country with a self-run IRB was the Research Institute for Tropical Medicine under the Department of Health (DOH) [7]. By the early 2000s, “only 50 percent of [Philippine] institutions [had] an [IRB].” [8] (p. 24).

In 2006, the Philippine Health Research Ethics Board (PHREB) was officially established. Consisting of members from various research disciplines, the board’s mandate includes overseeing the establishment and performance of IRBs in the country, as well as “[networking] with relevant local, national and international organizations.” [9] As of January 2021, the PHREB website lists 104 accredited IRBs in the country, 40 of which are found in the capital region of Metro Manila.

The country’s leading academic institutions established their own IRBs only within the last 15 years—for example, in 2010 for the University of the Philippines (UP) Manila, which houses the National Institutes of Health and is widely considered the country’s premiere health-sciences university [10]; and in 2015 for Ateneo de Manila University [11]. For the social sciences, the country’s mother organization, the Philippine Social Science Council, instituted its dedicated IRB only in 2017 [12].

Significantly, the Single Joint Research Ethics Board (SJREB) was formed under the DOH in 2017. Tailored specifically to ease the ethics approval process for multisited studies, the SJREB is the closest to a one-size-fits-all mechanism for multisited protocols in the country, in that it furnishes such protocols with blanket approval applicable to all hospitals under the DOH, allowing researches to proceed with data gathering without needing to obtain separate approval for each site [13].

## Methodology

From July to October 2020, we conducted semi-structured interviews with a total of 40 researchers in the Philippines. Because of the coronavirus disease 2019 (COVID-19) pandemic, we had to conduct the study remotely; at the time, our team members, all health researchers with experience in qualitative work, were each based in a different part of the country. Heeding Mays and Pope’s [14] call for purposive, theoretically informed sampling, and drawing on what Marshall describes as the “researchers’ practical knowledge of the research area,” [15] (p. 523) we purposively recruited our participants through peer referrals and targeted searches via Google Scholar, keeping the list as inclusive as possible so long as the interviewee was relevant to the ethics review process (e.g. as a researcher, board member, journal editor, government health official, hospital residency training officer). However, navigating the ‘new normal’ of the pandemic, and the changes it imposed upon academia and research culture, meant that realistically we had to simplify our participant selection and also consider the fact that our potential interviewees—all fellow researchers—were imaginably in similar straits with regards to adjusting to the pandemic. As such, for ease of access, we first approached individuals who were already known to us (e.g. at work), either through phone call, text message, or e-mail, before blindly contacting those with whom we were completely unacquainted. Prior to the interviews, our participants were sent the interview guides for their perusal; this preliminary correspondence also served as an avenue for them to raise questions about the study or concerns regarding their participation.

We initially categorized our participants according to three major disciplines: clinical research, public health research, and social science research. IRB members constituted a fourth category, and key informants—ranging from journal editors, heads of private research firms, to past and present government officials in health- and research-related fields—constituted the fifth. However, many participants did not necessarily belong to only one category, thus accounting for the participant distribution according to discipline exceeding the total number of actual participants. Save for three participants—two from the Northern Mindanao region and one from Cavite province—all interviewees were based in Metro Manila. Nearly half were affiliated with the UP system. Fifteen participants were early-career researchers (including graduate students), while the rest were considered established researchers. Data saturation became our determinant for the final sample size: During data gathering, our team regularly consulted with one another regarding the findings of an interview in order to determine whether we had attained some form of data saturation in each category of interviewees. Cognizant of our study’s aim to provide a preliminary—and not necessarily a definitive or exhaustive—picture of our subject matter, we would still encounter considerable saturation usually by the 10th interviewee, after which we would cap interviews for that category. In the end, our selection also considered a gender balance, workplace affiliation, and research discipline, all summarized in Table 1.

**Table 1:** Description of participant characteristics

Distribution according to gender	
Male	21
Female	19
Distribution according to research discipline	
Clinical research	13
Public health research	11
Social science research	14
IRB member	10
Key informant	5
Distribution according to affiliation	
University of the Philippines system	17
Private universities or teaching hospitals	10
Nongovernment research firms	9
Others	4

Our remote interviews were conducted either through phone call or video-conferencing software like Zoom. This capability to participate in a remote interview became our sole exclusion criterion. These interviews usually took 30 to 45 min. Although the interviews followed a general format—one that began with the interviewee’s personal experiences (e.g. their professional background, their individual experiences in research and with IRBs) before transitioning to more general topics like their views on ethical research, IRB practices in the country, and the impact of IRBs on research culture—we still tailored our questions according to the participant’s background and therefore used four separate guides (see Additional file 1 for the final interview guides). To reduce bias during the interviews, given that our team members all had prior interactions with IRBs, we strove as much as possible to follow the interview guide, sticking to one open-ended question at a time, and to remember that we were there as interviewers only, and not co-generators of insight. Our initial interviews served as pilot tests, but as we conducted more interviews, we also adjusted and tweaked specific questions that were inconspicuously biased, as well as allowed the participant’s response to shape succeeding or follow-up questions.

Consent forms were signed electronically; likewise, participant tokens were delivered via an online medium. The audio copies of the interviews were sent to transcribers who had signed nondisclosure agreements, and upon receiving the finished transcripts, our research team proceeded to ensure participant anonymity by removing as much identifying information as possible in each transcript. Only our research team has access to all 40 transcripts, which have been secured in a password-encrypted folder.

All transcripts were uploaded to a secure, offline NVivo 10.0 database and approached by way of deductive thematic analysis. Guided by our literature review, our research team first read the transcripts individually to come up with initial codes. We consulted with our team members regularly to ensure our individual readings of the text were devoid of researcher bias. Comparing codes led to our preliminary themes, after which we did another round of reading to arrive at the final themes by consensus. Our study was approved by the UP Manila Research Ethics Board (UPMREB 2019–259-01).

## Results

Across all three sectors of health research, researchers are one in recognizing the importance of ethics in the conduct of their work. All our clinical researchers, for example, agreed that it is now safer than ever to conduct trials precisely because IRBs exert rigid measures in evaluating protocols. But such procedural rigidity has also been criticized, with one participant describing the whole application process as “intimidating.” In this section, we discuss these criticisms and offer perspectives from researchers who are also IRB members, with particular attention to the differences among the three sectors.

### Ethics review takes a long time

The researchers’ most common and prominent complaint is that the review process in the Philippines is time-consuming. According to them, it takes anywhere between two weeks to a month from the time of protocol submission before IRBs even give an initial decision, and between two to three months before a final decision can be reached. Accounting for protocol revisions and resubmissions, the whole process can sometimes last as long as “almost a year,” disrupting research timelines even when a long process has been anticipated. A public health researcher said a project she was involved in was delayed for almost a year only because the protocol was not approved immediately—despite zero requests for revisions from the reviewers. Separately, a consultant at a tertiary training hospital shared:

Our hospital was supposed to participate in an international clinical trial. But it took a long time for the ethics board to approve the study that by the time we got approval, the trial was about to close. We ended up withdrawing our participation.

As the latter quote suggests, the time-consuming nature of the review process is of great concern particularly for researchers who are working on urgent studies or operating on a limited timeline. A head of a private research firm counted at least three recent instances of potential international collaborators backing out of a Philippines-based study for this reason alone. Even non-professional settings experience similar constraints: A university professor talked of instances when students got delayed or had to drop out simply because their thesis proposals were not approved promptly. Consequently, researchers are either forced to meticulously account for the estimated review duration in structuring their studies—or altogether abandon certain aspects of proposed projects, if not their entirety, that cannot afford longer time frames.

Another impact is that, within the health research community in the Philippines, certain IRBs have gained reputations in terms of how fast they can process applications. Our participants spoke of knowing exactly which boards have fast turnaround times and can be relied upon to approve studies with limited timelines. One researcher described an IRB based in a private hospital as almost “machine-like” in its predictability to approve a study swiftly, making it a favorite among those in a hurry despite being “less prestigious” than university-based boards.

Our participants did identify experiences with efficient IRBs that can produce initial results within two weeks and finish the whole process in a month. Some also said the COVID-19 pandemic has forced IRBs to be more efficient—for instance, by finally accepting email submissions and carrying out correspondences accordingly. Nevertheless, on the whole, the researchers felt that IRBs don’t expedite reviews as often as they should or provide more manageable timelines.

### Ethics review is costly

Regarding the financial aspect of applying for ethics approval, most of our participants said that the cost is usually not a problem—so long as the concerned study has funding. That said, our interviews showed notable variability in the rates that IRBs charge per application. Some university boards, for example, completely waive their fees for all student and faculty applications, while others provide considerable discounts. On average, however, the fees range between PHP 20,000–50,000 (US$ 416–1,040), and can go as steep as PHP 80,000 (US$ 1,664) per application.

Even with funding, our participants admitted to being concerned at how these rates eat up a considerable chunk of the budget. But for independent and student researchers, the grim and obvious implication is that these fees must come from their own pockets—leading one public health researcher to speculate that the barrier inevitably imposed by these fees may not only be limiting the country’s research landscape to certain circles where funding is easily available (e.g. academe), but may also be discouraging independent or starting researchers from pursuing their work. That participant continued:

As head of [redacted private research firm], even I find a fee of PHP 30,000 [US$ 624] exorbitant. But I’ve also been approached by friends from abroad, PhD students who want to do research in the Philippines, who are just shocked at how much our ethics boards want to charge them.

Moreover, precisely because there is no rigid regulation enforcing some semblance of uniformity upon IRB rates, some participants were concerned that IRBs have been taking advantage of this “financial opportunity,” as this public health researcher illustrated:

The bill for ethics review can include two items. One is the ‘ethics review fee’, which is manageable, say, at around PHP 20,000 (US$ 416). But a lot of hospital boards add the second item, a so-called institutional fee—and that’s where it gets super arbitrary. For a project that I did with an American government agency, for example, one of our hospital sites charged us something close to PHP 100,000 [US$ 2,081], which was way above what we could have expected. When we asked for justification for the fee, they couldn’t even provide one, which to us was code for, ‘We know you have a lot of money, so cough it up’. It’s ironic that so-called ethics boards engage in such a gray area of practice.

As that excerpt shows, the apparent inconsistencies in and unpredictability of these fees are worrying enough—but more troubling is the idea that ethics review is fast becoming a business. One social scientist said it best:

I’m convinced there is a market for ethics review—that it can be a lucrative industry if you want to go down that path. I mean, reviewers just have to read a protocol, comment on it, eventually give a decision—and the board can earn something like PHP 50,000 [US$ 1,040] from it? And if they’re fast, it will only take a month to do that, even less.

That, in fact, is precisely what a member of a private hospital’s in-house board confided:

It’s easy to say that having their own in-house IRB can position hospitals as prime movers and innovators when it comes to research. But an IRB is also admittedly a huge source of funds. There is so much money [to be earned from participating] in multi-country clinical drug trials. Coming from a private-hospital standpoint, I’d say that is one very compelling reason to form an IRB.

### Ethics committees are concentrated in major cities

Aside from the time and cost, our participants also identified the concentration of IRBs in Metro Manila as a concern, particularly for researchers based in the provinces. A clinical researcher from a major Northern Mindanao city, for example, said that in her city, she “wouldn’t know of any other ethics board to go to besides the one in my hospital.” Likewise, a social scientist from a neighboring town said that, as far as his field of research is concerned, he always has to go “out of town” to get ethics approval.

Many researchers agreed that the SJREB has been a welcome solution for multisited studies. But, as one researcher from the DOH noted, this solution has its own shortcomings: “One of the earlier challenges [encountered by SJREB] was resistance from the individual ethics review boards, who felt like they were surrendering their power and feared they would not be paid anymore individually.” Another public health researcher pointed out a second shortcoming of this system: Since the SJREB strictly applies only to hospitals under the DOH, private institutions can refuse to recognize that blanket approval and still require researchers to apply for separate approval under their own boards (as was this participant’s experience). “Instead of going through one board [SJREB only], in the span of a year our team ended up going through three [including two private institutions that acted as described],” that participant said. Thus, up to now, the need to obtain ethics approval remains a preliminary obstacle among those whose institutions—or cities—do not have their own IRBs.

### Ethics review is designed for clinical research

Non-clinical researchers highlighted a pressing concern not shared by their clinical counterparts: Their perception that IRB processes have been designed with clinical research in mind—and therefore inappropriate for non-clinical disciplines. Fundamentally, this clinical orientation manifests in the paperwork that IRBs require researchers to accomplish. As a public health researcher said:

It’s bad enough that [IRBs] that have yet to transition to digital require you to fill out a ton of paperwork. But many of those questions and forms are actually irrelevant to non-clinical studies. It would be more efficient for everyone if, for example, template forms were already designed according to specific types of studies.

This bias is also reflected in IRBs’ compositions—and the kind of mindset and expertise, or lack thereof, that members consequently bring into their work. For example, one social scientist pointed out how hospital-based IRBs are usually composed of clinicians—and therefore

cannot be expected to competently evaluate social science researches... [Moreover,] it’s not just a matter of board composition—a certain sector being overrepresented or underrepresented—but also a matter of lack of training. Boards in the Philippines just aren’t as interdisciplinary as they ought to be.

As such, to quote a clinician who does mostly qualitative research:

Ethics boards can disapprove or question protocols not because they are ethically unsound, but because they have subjective qualms over methodology—and most of the time these qualms result from the simple fact that the board members are unfamiliar with how that methodology works.

What happens in such cases, a participant from a firm that specializes in outsourced processing of hospital-based protocols said, is that IRBs then tend to act in a reactionary manner. “They react to what you show them rather than knowing the right questions to ask. I’ve handled many studies with novel or unfamiliar data-collection tools that got questioned relentlessly over their design alone.”

It is easy to see, then, where the complaints regarding IRBs overstepping their mandate or “meddling with the study,” to use one participant’s words, are partly rooted on—and why, when IRBs justify such 'overreach' by saying that “a study cannot be ethically sound if it is not methodologically or technically sound,” researchers would not believe them. Across all three sectors, we had participants who raised that exact point, identifying the frequent overreach of authority on the part of IRBs as a major cause of tension between researchers and boards. This tension becomes more discernible as one deviates farther from clinical research and is most pronounced in the social sciences.

More than one social scientist experienced working with a board that insisted on written informed consent for non-clinical studies on vulnerable populations where non-written consent has long been established as the safer practice. In one such study, the board refused to back down, giving the researcher no choice but to forego the project. One researcher working on an ethnographic study with indigenous peoples—a field where study populations are now recognized as co-generators and co-owners of the data yielded during fieldwork—related the difficulty of convincing an IRB that the biomedical norm of destroying data after a certain time was not applicable in this case. A university professor shared how an IRB once refused to approve a thesis advisee’s proposal not on ethical concerns, but on objections to the methodology:

It was an online survey with nothing particularly sensitive about it—I should know because I had already vetted it—so after all the back-and-forth with the board, I put my foot down and sent them a stern letter telling them to stop messing with our methodology. They backed down and let the study proceed as originally designed.

### Ethics review for ethics review’s sake?

Taken together, the above complaints shape Filipino researchers’ attitudes toward the ethics approval process—and influence decisions on which topics to do research on, which IRBs to apply to, and whether to pursue a research idea in the first place. As one social scientist confessed:

It quickly becomes discouraging to do research in the country because your mindset, as far as IRBs are concerned, is that you will really have to fight to get approval. I have had experiments in mind that I’ve had to abandon over the mere thought that I wouldn’t get approved.

Given how the ethics review landscape is only in its relative infancy, the same participant continued, “the way these boards are operating right now, it’s almost like they think they’re at a dissertation defense and researchers are there to defend their study.”

“Here in the Philippines it’s almost as if IRBs are dictating to researchers, treating them like they don’t know any better,” said the head of a private research firm, going on to say how

it becomes difficult to accept that kind of treatment when you know these boards are not perfect themselves. The worst experience I’ve had was when a study was returned to me after quite some time with comments that had absolutely nothing to do with my paper—because they were meant for another study.

Such experiences have led researchers to adopt a pragmatic view of ethics: as a necessary, if bureaucratic, step in their projects. “We only look at two things,” a private-sector researcher said, “fast turnaround times and inexpensive costs.”

A public health researcher separately added that the ethics review process is all about working toward an approval, and the moment that approval is obtained, the whole process abruptly comes to an end: There is usually no active follow-up from the IRBs during the course of fieldwork and beyond, even as the requirements for approval emphasize the necessity of such a process; in fact, most of the time, the burden of follow-up falls on the researchers themselves, who end up actively updating the boards regarding their progress. In this sense, ethics approval is reduced to a mere piece of paper, something to be obtained, rather than a thorough evaluation of the conduct and impact of the study both from within and without. In the end, that same researcher said, the demands of IRB application processes take away time that would have been better devoted to preparing for the actual analysis, writing, and publication of a study.

In fact, some participants fear that ethics boards may be fomenting a culture of gatekeeping insofar as research is concerned, given their lack of accountability. Despite the existence of PHREB, these researchers complain that there is no feedback mechanism to air their concerns regarding the approval process and no discernible metric system to evaluate the performance of IRBs.

In many ways, all these criticisms are reflective not only of IRB practices in the country, but of the larger research landscape. Commenting on the inordinate amount of time it takes IRBs to process applications, a participant from a firm specializing in outsourced processing of hospital-based protocols said:

The perfect way to describe our research landscape is that there is a lot of research waste going on, and it’s all rooted in the lack of protected time for research. I’ve seen how it is in other countries, where even junior researchers are really given protected time to do research, where the whole system is very accessible. Here, the fact that we don’t have such dedicated time already affects the quality of writing in our protocols to begin with.

“Ethics should be ingrained in every researcher and must go beyond the IRB,” said a social scientist. At the very least, that same scientist said,

IRBs should place a certain level of trust on the researchers they are working with or the technical review boards who have separately screened the protocols... Right now, how IRBs are shaping the way we do research is in the mode of ‘I'll develop a research protocol that will be so benign, it will be immediately approved by an ethics committee’, instead of ‘I will be making a protocol that will not affect the lives, health, and well-being of my respondents’. Those are two completely different things.

### Counterpoint: perspectives of IRB members

From the standpoint of researchers who have worked in or are currently members of IRBs, the logistical limitations of IRBs can be explained by the simple fact that unlike other countries, the Philippines has yet to professionalize research ethics. One participant said one of the birthing pains of “building a culture of research ethics” is getting people to appreciate and participate in research ethics, to serve as reviewers and panel members. A participant from the DOH observed how “most hospitals have no actual budget dedicated to sustaining an ethics board. That is why ethics boards become highly dependent on the fees that they charge to, say, pay for the office space, the secretariat and administrative staff, etc.” As a clinician-researcher put it:

The operation of IRBs is governed by a lot of privacy and confidentiality. But even in big universities, we are not always assured of lockable cabinets. We should not be sharing a fax machine with another office. We should have our own shredder.

Protracted turnaround times can be easily explained by the fact that IRBs are often understaffed and swamped with protocols. Almost always, IRB members also have other responsibilities, such as being university professors or clinicians, and cannot devote all of their working time to just evaluating protocols. Almost always, as well, the compensation for IRB members is hardly commensurate to the amount of time and effort that they devote to the work. One participant described present conditions succinctly:

Reviewers are asked to read 300-page protocols and paid for only an hour of the job. So you really have to question why, in a clinical trial, for example, an IRB would charge PHP 60,000–70,000 [US$ 1,250–1,455] for review but pay its reviewers only PHP 1,000–2,000 [US$ 20–42] per protocol reviewed.

Where the perspectives of IRB members diverge from our other participants is in the mandate of an IRB. “Many researchers cling to this belief that ethics boards are barriers to research,” said one board member. “Researchers tend to become preoccupied with the science of their protocols and end up ignoring the ethics of their protocols.”

According to this subset of participants, a lot of the back-and-forth that researchers complain about in their dealings with IRBs has to do with the simple fact that these protocols tend to be badly written. Researchers can overlook the major, complicated issues such as the study design, but, as one participant noted, the oversight can be far simpler: “I have received studies that, in the objectives, stated the intent to compare this and that, but this intent to compare is not even reflected at all in the study design.”

They also emphasize that the existence of technical review boards is not an excuse for IRBs to be less strict. According to one participant,

preliminary technical review is helpful, but in my experience, most of the time it pays for me as an ethics reviewer to also assess the technical aspects simply because the technical review can miss out on a lot of things that then make the study ethically unsound.

Ideally, that participant continued, if the finances and manpower can make it possible, only one committee should do both ethical and technical reviews to streamline the whole process. In the end, another participant said,

researchers don’t realize that when an IRB ‘oversteps’ and points out technical issues, it’s not because of overreach or a matter of gatekeeping. It’s because as an IRB member, you realize that the study goes beyond the researcher; it will affect individuals and communities.

A social scientist belonging to an IRB provided this illustrative summary to the argument:

Let’s say you are doing a study on the impact of COVID to mental health. If you settle for 400 as your study population, imagine asking 400 people these intrusive questions that would only magnify the risks that they may have already been exposed to. Inserting ethics into the question makes you ask, for example, whether you can achieve the same results with half the population size. It makes you think about the responsibilities of a researcher, so before you submit your protocol for approval, you are already considering the soundness of the design and other questions that you would have otherwise ignored if you were solely focused on the science.

## Discussion

### An issue of inequity

On the whole, our study resonates with the global literature that have examined—and/or have been critical of—the role, culture, and practices of research ethics committees, beginning with an acknowledgment of their necessity: Faced with the prospect of ethical review, most of our participants echoed Schrag’s observation, in that their first instinct is generally one of “eager cooperation.” [16] (p. 122)

But as our findings also showed, the rift between the perspectives of researchers and IRBs regarding the review process transcends mere diverging views on what constitutes “ethical research.” And in any case, the problems raised by Filipino researchers regarding that process are only part of the continuum of ongoing debates worldwide—from the time-consuming and paperwork-heavy nature of ethics approval described by Gold and Dewa [17] in their study of multisited researches; imbalances in board composition that, as Schuppli and Fraser [18] wrote, render evaluations ineffective; and the highly clinical framework of current review processes that Flicker et al. have criticized for possibly doing more harm than good and “placing communities at risk” through an insistence on inappropriate measures of evaluation, particularly as imposed upon the social sciences and participatory research [19]. To go by Abbott and Grady, the Philippine situation is similarly riddled with all forms of inefficiencies and inconsistencies, be it in terms of application fees, duration of review, or outcomes of evaluations [20]. Additionally, although our limited findings disallow us from fully arriving at the same, incontrovertible conclusion, the anecdotes of our participants regarding commercial IRBs nonetheless find some measure of resonance in Lemmens and Freedman [21], who, a full two decades earlier, already voiced concerns regarding profit-related conflicts of interests as regards the operation of these ethics boards, and how these conflicts may eventually compromise the quality of review and erode public trust in IRBs.

In its most fundamental sense, then, ethics review is an issue of inequity. The existing system serves as yet another barrier for those outside the academe; those belonging to institutions, cities, or entire regions without their own accredited IRBs; and researchers working independently, without ample budget, or on highly specialized topics. And even for researchers affiliated with institutions that have their own IRBs, the balance invariably tips toward those with sufficient funding and/or those working on topics that tend to receive funding. All of these contribute to the rise of what Patterson [22] calls “spaces of marginalization” that privilege certain types of research, and research topics—and eventually, knowledge production—over others.

As our own research demonstrates, non-clinical researchers find themselves at the marginal end of that divide. For these researchers, the inequitable landscape is also one where they must abide by what Schrag describes as “silly restrictions” that are inapplicable to their respective fields, and which are imposed mostly by IRBs that lack the expertise to properly evaluate protocols and end up applying “inappropriate” principles to such evaluations [16]. Often, IRBs operating on a highly biomedical framework have exaggerated “protectionist concerns,” [23] (p. 483) the imposition of which do not necessarily result in more ethical research practices [24]. Instead, in such a landscape, current guidelines may only serve to actually “[impede] ethically sound or potentially beneficial research,” [25] (p. 161) and may be entirely unable to address more complex, field-related situations already raised by existing scholarship [26]—further straining researcher-IRB relations and aggravating researchers’ feelings of mistrust, if not outright antagonism [27, 28]. At worst, this “unquestioning transposition of ethical principles from [clinical to non-clinical] research [may] lead to inappropriate practices that… actually encourage *less* ethical practice.” [24] (pp. 94–95).

Ultimately the demands that the ethics application process imposes upon researchers cannot simply be reduced to discrete complaints—on matters of time, or finances, or geographical proximity, or expertise from the side of IRBs; it is an interplay of these factors that breeds an environment that favors those with the resources to do research—and those with researches that are more likely to attract funding. In this manner, indeed, the ethics review process becomes a matter of (in)equity.

### Recommendations

While the broader challenge of equity in health research will entail more fundamental reforms (as detailed by Pratt, Merritt and Hyder [29]), more proximal interventions can be done to improve the research ethics process and make it more equitable. For instance, an oversight committee—in the case of the Philippines, PHREB—should take a more proactive role in mediating the debates of research culture, and go far and beyond its mandate to promote the proliferation and evaluate the performance of IRBs—with special emphasis on what Coleman and Bouësseau [30] term outcomes assessment, or ensuring from the IRBs’ end that so-called ethical research actually carries on to the field. This is not to undermine the trust between researchers and IRBs once a research has been approved, but to say that IRBs that require researchers to submit regular updates during data gathering, for example, should also be more proactive in seeking those updates or tackling unforeseen ethical dilemmas that may arise *after* protocol review. Else, what persists is the image of ethics approval as the ‘golden calf’ of the research process, a mere objective to be attained and surmounted.

Moreover, such a committee could also act as a mediator in the financial burden of ethics review—for example, by regulating and imposing uniformity on fees—thereby potentially eliminating conflicts of interest where money and/or power relations is concerned, making ethics approval more equitable while still allowing IRBs a comfortable measure of financial self-sufficiency (see, for example, the arguments of Emanuel, Lemmens and Elliot [31]). That the complaints of our participants who have worked or are part of IRBs centered mostly around the lack of material support (in terms of personnel, office resources, and/or protected, compensated time for IRB-related duties) only makes this kind of financial self-sufficiency an imperative—and all the more so when one considers how this problem has been perennially recognized across global literature [20, 32].

For researchers, the institutions they represent, and IRBs, the spirit of equity should extend to supporting research topics that have not enjoyed as much scholarly attention and making the landscape more hospitable for early-career, student, and independent researchers. To paraphrase Chatfield et al. [33], making this possible includes ensuring that IRBs are staffed with the right kind of ‘qualified experts’—given that there are “no absolute standards upon which IRBs can rely [in evaluating protocols]” and ethics boards must therefore bank on “a fair exercise of intelligence and discretion on the part of [their] members” [21] (p. 562)—and lessening the bureaucratic roadblocks that make even getting ethics approval already a laborious process. To recapitulate a point made earlier, a change in research culture requires *not* taking time away from the actual conduct of the study—from data gathering, analysis, and paper writing. In other words, fostering “a culture of ethics” instead of “a culture of red tape,” to quote Burris and Welsh in Coleman and Bouësseau [30] (p. 4).

Doing so eventually boils down to the kind of larger research culture that we foster, where: (1) research is seen as an integral part of knowledge production, in the academe as in other fields of society, and therefore granted protected time and sufficient funding; (2) IRBs are seen as instrumental partners in research and therefore granted sufficient human, material, and financial resources to fulfill their mandate; (3) “ethical research” is not viewed as culminating in IRB approval, but as foundational to research—and practiced deliberately, from the writing of the study protocol to the final study analysis; and (4) a certain level of ‘trust’ is established between researchers and IRBs [34, 35], manifesting not through what Makhoul [36] aptly labels “policing,” but through a “collaborative and supportive relationship” that makes ‘allies’ out of IRBs and researchers [32].

## Conclusion

Our study has certain limitations. First of all, our limited sample size means that our findings cannot be interpreted as unequivocal generalizations of the health research landscape in the Philippines, or even as definitive pictures of each sub-field of health research as far as IRBs are concerned. Moreover, almost all of our participants hailed from Metro Manila, making it impossible to account for specific experiences in the country’s many other cities and regions, given the variability in academic and research culture across the nation. Our participants’ affiliations were likewise limiting: We had no interviewees who were undergraduate students, for instance, and nearly half of our participants were affiliated with the state-funded UP system—a fact that would have no doubt colored our participants’ careers and narratives. The focus of our study itself—IRBs in the context of health research—disallows our findings from being considered as entirely valid conclusions for other fields of academic, and even non-academic, research in the country. As such, future avenues for research may include IRB culture and practices in non-health research, as well as the relative infancy of multisited common review in the country.

Nevertheless, our study shows how the case of the Philippines can serve as a point of reflection for similar low- and middle-income countries where research inequities have persisted. The narratives of our participants show how, despite growing recognition of the fundamental role of IRBs to research ethics, the research landscape remains one that is riddled with structural biases and deficiencies—problems that are challenging to overcome, but which are definitely not without solutions. Arguing that ethics review is *essential* even for non-medical research, Lindoff [37] calls for opening up lines of dialogue between researchers and IRBs, highlighting the importance of such partnership between the two parties. To this, we concur—but with the caveat that such a partnership may flourish only when the ethics approval process ceases to be a barrier for researchers. Should a middle ground exist, it should be one where researchers need not surmount numerous structural inequities even before a study has commenced; one where researchers don’t have to “fight” for ethics approval.

## Supplementary Information

**Additional file 1**. Interview guides tailored according to participant background (e.g. researcher, ethics board member, hospital administrator, etc.).

## Data Availability

Data sharing is not applicable to this article, which is grounded on qualitative interviews with key informants.

## Abbreviations

COVID-19: Coronavirus disease 2019
DOH: Department of Health
IRB: Institutional review board
PHREB: Philippine Health Research Ethics Board
SJREB: Single Joint Research Ethics Board
UP: University of the Philippines
