# Supplementary material for: How ethics committees and requirements are structuring health research in the Philippines: a qualitative study
Source: BMC Med Ethics. 2021 Jul 1;22:85. doi: 10.1186/s12910-021-00653-z (PMC8246435; doi:10.1186/s12910-021-00653-z)
Supplement: Supplementary file 1 — Additional file 1. Interview guides tailored according to participant background (e.g. researcher, ethics board member, hospital administrator, etc.). [file 12910_2021_653_MOESM1_ESM.pdf]

<u>Additional file 1: Interview guides</u>

1. Administrators (Heads of research firms, hospital directors, etc.)

**Questions**	**Notes**
1. Tell us about your work	Ask the participant to narrate tasks, experiences, challenges
2. How does ethics figure in your work?	- Do you require ethics approval in the projects of your staff/ faculty/ researchers?<br>- Are there exemptions?<br>- Elicit interesting cases in the past, if any
3. What difficulties do researchers face in terms of securing ethics?	- Requirements and paperwork<br>- Time and budget constraints<br>- Reviewers' responses<br>- Methodological adjustments, if any
4. What do you feel about ethics boards in the Philippines?	- Participants' perceptions on the whole idea of research ethics<br>- Notions of change over the years<br>- Methodological challenges
5. In your experience as a researcher and in dealing with researchers, how important is ethics, and ethics approval, as a consideration in your/the research?	- Impact on choice of topics<br>- Impact on choice of methods<br>- Impact on budget, time, and other elements in research proposal
6. What are the differences in the ethics policies of various institutions? How would you compare and contrast them?	- Best practices<br>- Negative experiences<br>- Global and national standards
7. If you have the power to change the ethics approval process, what would you do?	- Recommendations to improve ethics processes in the Philippines

2. Ethics Board Members

**Questions**	**Notes**
1. Tell us about your work as a member of the ethics boards. How long have you been involved?	Ask the participant to narrate tasks, experiences, challenges
2. Can you tell us a brief background of the ethics board itself?	Ask the participant to recount the institutional history of the board - what was the rationale for its creation, what were the challenges, etc?
3. Please tell us about the process of ethics review	How long does it take? (Elicit time to first feedback and time to final approval)<br>- Do researchers have to pay for it? (If so, how much?)<br>- What about reviewers? How are they selected, and how do they get paid?
4. What difficulties do researchers face in applying for ethics?	- Requirements and paperwork<br>- Time and budget constraints<br>- Reviewers' responses<br>- Methodological adjustments, if any
5. What difficulties do ethics boards face in doing their work?	- Bureaucratic challenges, dealing with re- searchers, reviewers, DOH, etc.
6. What do you feel about ethics boards in the Philippines?	- Participants' perceptions on the whole idea of research ethics<br>- Notions of change over the years<br>- Methodological challenges
7. What are the differences in the ethics boards of various countries and institutions? How would you compare and contrast them?	- Best practices<br>- Negative experiences<br>- Instances where same project needs more than one approval
8. If you have the power to change the ethics approval process, what would you do?	-Recommendations to improve ethics processes in the Philippines

3. Journal Editors

Questions	Notes
1. Tell us about your work as part of the journal	Ask the participant to narrate tasks, experiences, challenges
2. How does ethics figure in your work?	- Do you require articles to have ethics approval?<br>- Are there exemptions?<br>- Elicit interesting cases in the past, if any
3. What difficulties do researchers face in terms of securing ethics?	- Requirements and paperwork<br>- Time and budget constraints<br>- Reviewers' responses<br>- Methodological adjustments, if any
4. What do you feel about ethics boards in the Philippines?	- Participants' perceptions on the whole idea of research ethics<br>- Notions of change over the years<br>- Methodological challenges
5. In your experience as a researcher and in dealing with researchers, how important is ethics, and ethics approval, as a consideration in your/the research?	- Impact on choice of topics<br>- Impact on choice of methods<br>- Impact on budget, time, and other elements in research proposal
6. What are the differences in the ethics policies of various journals? How would you compare and contrast them?	- Best practices<br>- Negative experiences<br>- Global and national standards
7. If you have the power to change the ethics approval process, what would you do?	-Recommendations to improve ethics processes in the Philippines

4. Researchers

Questions	Notes
1. Tell us about your research interests and research projects	Ask the participant to enumerate their completed or ongoing research projects
2. Which of your researches did you apply ethics approval for? Why did you (not) apply for ethics approval for those particular researches?	- Was it required by your institution?<br>- Was it required by the funder or international partner?<br>- Concerns over publication
3. For those you applied ethics approval for, please tell us about the process of applying	- Where did you apply? (Why?)<br>- How long did it take? (Elicit time to first feedback and time to final approval)<br>- Did you have to pay for it? (If so, how much?)
4. What difficulties did you face in applying for ethics?	- Requirements and paperwork<br>- Time and budget constraints<br>- Reviewers' responses<br>- Methodological adjustments, if any
5. What do you feel about ethics boards in the Philippines?	- Perceptions of the whole idea of research ethics<br>- Notions of change over the years<br>- Methodological challenges
6. How important is ethics, and ethics approval, as a consideration in your research?	- Impact on choice of topics/ choice of methods<br>- Impact on budget, time, and other elements in research proposal
7. What are the differences in the ethics boards of various countries and institutions? How would you compare and contrast them?	- Best practices<br>- Negative experiences<br>- Instances where same project needs more than one approval
8. If you have the power to change the ethics approval process, what would you do?	- Recommendations to improve ethics processes in the Philippines
